# Peritraumatic distress fully mediates the relationship between posttraumatic stress symptoms preoperative and three months postoperative in patients undergoing spine surgery

**DOI:** 10.1080/20008198.2018.1423824

**Published:** 2018-01-19

**Authors:** Ehab Shiban, Jens Lehmberg, Ute Hoffmann, Jeff Thiel, Thomas Probst, Margret Friedl, Andreas Mühlberger, Bernhard Meyer, Youssef Shiban

**Affiliations:** ^a^ Department of Neurosurgery, Technical University of Munich, Munich, Germany; ^b^ Department for Psychotherapy and Biopsychosocial Health, Danube University Krems, Krems, Austria; ^c^ Department of Psychology (Clinical Psychology and Psychotherapy), University of Regensburg, Regensburg, Germany

**Keywords:** Posttraumatic stress disorder, risk factors, peritraumatic distress, elective spine surgery, mediation analysis, Trastorno de estrés postraumático, factores de riesgo, estrés peritraumático, cirugía espinal selectiva, análisis de la mediación, 创伤后应激障碍, 危险因素, 围创伤应激, 选择性脊柱手术, 中介分析, • Illnesses and surgical procedures are potential risk factors for PTSD.• The relation between pretraumatic PTSD symptoms, peritraumatic distress and posttraumatic PTSD symptoms among elective spine surgery patients is investigated.• Peritraumatic distress plays an important role in the development of PTSD symptoms.• Psychological treatment of patients at risk of developing PTSD can be beneficial to ensure both mental health and optimal recovery from surgery.

## Abstract

**Background**: Growing evidence shows the significance of illness and surgical procedures as traumatizing stressors. Risk factors are widely investigated in various settings and samples, using numerous measures of posttraumatic stress and posttraumatic stress disorder (PTSD). While pretrauma psychological distress is acknowledged as an influential factor, peritraumatic experiences are controversially still being discussed as relevant to the development of PTSD.

**Objective**: In a group of patients consecutively undergoing elective spine surgery (*N* = 89) in a German hospital, this longitudinal study addressed the question of how pretrauma PTSD symptoms and peritrauma distress interact with one another in regard to the amount of posttrauma symptoms of PTSD.

**Methods**: Pre- and posttrauma symptoms of PTSD as well as peritrauma distress were assessed through questionnaires one week before, one week after or three months after surgery.

**Results**: Even though all three variables showed significant correlations with one another, mediation analysis revealed that peritrauma distress fully mediated the relationship between pre- and posttrauma PTSD symptoms.

**Conclusions**: These results add new insights to the controversial discussion on the role peritraumatic experiences play in the development of PTSD, especially in medical settings.

## Abstract (es)

**Contexto**: Evidencias crecientes muestran el significado de la enfermedad y de los procedimientos quirúrgicos como estresores traumatizantes en si mismos. Los factores de riesgo son ampliamente investigados en varios contextos y muestras, usando numerosas medidas de estrés postraumático y del trastorno de estrés postraumático (TEPT). Mientras que el estrés psíquico pretraumático es reconocido como un factor influyente, las experiencias peritraumáticas están siendo aun objeto de controversia como elementos relevantes para el desarrollo de un TEPT.

**Objetivo**: En un grupo de pacientes sometidos a cirugía selectiva espinal (N=89) en un hospital alemán, este estudio longitudinal iba dirigido a estudiar la cuestión de en qué medida tanto los síntomas de TEPT pretraumáticos como el estrés pretraumático, interactúan uno con el otro en relación con la aparición de síntomas postraumáticos de un TEPT.

**Métodos**: Los síntomas pre y postraumáticos de TEPT así como el estrés pretraumático fueron evaluados y determinados a través de cuestionarios, una semana antes, una después y tres meses después de la intervención quirúrgica.

**Resultados**: Aunque las tres variables mostraron correlaciones significativas entre sí, el análisis de la mediación revela que el estrés peritraumático es el mayor mediador de la relación entre síntomas de TEPT, pre y postraumáticos.

**Conclusiones**: estos resultados añaden nuevas perspectivas a la controvertida discusión sobre el papel que juegan las experiencias peritraumáticas en el desarrollo del TEPT, especialmente en contextos médicos.

## Abstract (zh)

**背景**: 疾病和手术过程作为创伤应激源的证据越来越多。使用大量的创伤后应激和创伤后应激障碍（PTSD）测量，风险因素在不同的场景和样本中被广泛考察。

**目标**: 在德国一家医院中，选择一组持续经历选择性脊柱手术的病人（N=89）。本追踪研究试图回答创伤前的PTSD 症状和围创伤期应激如何相互影响对应阶段的PTSD 症状。

**方法**: 在手术一周前、一周后和三月后，使用问卷评估创伤前和创伤后的 PTSD 症状，以及围创伤应激。

**结果**: 尽管所有三个变量都显示出显著的相互关联，中介分析发现围创伤应激完全中介了创伤前后创伤后的 PTSD 症状之间的关系。

**结论**: 研究结果为关于围创伤经验在 PTSD 发展中的作用的争议提供了新视角，尤其是在医疗情景下。

## 1. Background

One important change in the fourth edition of the Diagnostic and Statistical Manual of Mental Disorders (DSM-IV) (American Psychiatric Association, 1994) was the inclusion of serious medical conditions as possibly traumatizing events. Since then, symptoms of posttraumatic stress disorder (PTSD) – such as awareness under anaesthesia (Osterman, Hopper, Heran, Keane, & van der Kolk, 2001), intensive care unit survivors (Davydow, Gifford, Desai, Needham, & Bienvenu, 2008), elective lumbar spinal arthrodesis (Deisseroth & Hart, 2012) and breast cancer (Mehnert & Koch, 2007) – have been investigated in various medical settings.

Prevalence of PTSD differs strongly among populations, types of trauma and measures (Brewin, Andrews, & Valentine, 2000). According to the DSM-5 (American Psychiatric Association, 2013), the projected lifetime risk for PTSD is 8.7% and the 12-month prevalence is 3.5% in the general US population, whereas lower estimates, around 0.5–1%, can be found in Europe. Numbers found in medical settings can vary considerably; for example, an overall incidence of PTSD symptoms following elective lumbar spinal arthrodesis was reported in 19% of all patients when including various postoperative time points (Deisseroth & Hart, 2012).

Even subclinical symptoms of PTSD, not just a fully developed disorder, have a notable impact on clinical outcome (Hart, Perry, Hiratzka, Kane, & Deisseroth, 2013; Tedstone & Tarrier, 2003). Therefore, it is crucial to investigate the emergence and course of those symptoms. Studies on the development of PTSD in medical settings have been mostly restricted to life-threatening conditions, such as myocardial infarction and intracranial bleeding due to an aneurysm rupture (Hutter & Kreitschmann-Andermahr, 2014; Visser-Meily et al., 2013; Wasson, Shaffer, Alcantara, Schwartz, & Edmondson, 2014). Because these conditions represent medical emergencies, it was impossible to conduct a baseline (pretrauma) psychological evaluation of these patients.

Although 60% of men and 50% of women (Kessler, Sonnega, Bromet, Hughes, & Nelson, 1995) in the general population experience life events that qualify as traumatic according to the DSM-III-R, only 24% (Breslau, Davis, Andreski, & Peterson, 1991) of those cases develop PTSD. Variables modifying the risk of developing posttraumatic stress can be classified as pretrauma, peritrauma or posttrauma variables (Schnurr, Lunney, & Sengupta, 2004). In meta-analyses, peritrauma and posttrauma variables had a higher predictive value of posttraumatic stress than pretrauma variables (Brewin et al., 2000; Ozer, Best, Lipsey, & Weiss, 2003; Trickey, Siddaway, Meiser-Stedman, Serpell, & Field, 2012).

Recently, pretrauma risk variables were classified into six categories: (1) cognitive abilities; (2) coping and response styles; (3) personality factors; (4) psychopathology; (5) psychophysiological factors; and (6) socio-economic factors (DiGangi et al., 2013). Kessler et al. (2014) analysed the predictive value of pretrauma variables in a large study involving individuals from 24 countries and found that posttraumatic stress symptomatology prior to exposure to a new traumatic event was the most powerful predictor.

Among the peritrauma variables found to be predictive of posttraumatic stress are perceived life threat during the trauma, peritraumatic emotional responses and peritraumatic dissociation (Ozer et al., 2003). Although peritraumatic dissociation attained the highest effect size (*r* = .35) in the meta-analysis performed by Ozer et al. (2003), more recent findings question whether peritraumatic dissociation can be considered an independent predictor (Hagenaars, van Minnen, & Hoogduin, 2007; van der Velden & Wittmann, 2008). Peritraumatic dissociation may rather be an epiphenomenon of high levels of peritraumatic distress (Fikretoglu et al., 2006). In addition to pretrauma and peritrauma variables, variables occurring after the traumatic event (posttraumatic variables, e.g. social support and life events) are associated with later posttraumatic stress (Brewin et al., 2000; Ozer et al., 2003).

Although pre-, peri- and posttrauma variables were found to be predictive of posttraumatic stress, the way they interact with each other often remains unclear. Certain pretrauma variables might influence posttraumatic stress directly, while others could influence posttraumatic stress indirectly through peritrauma variables. Peritrauma variables, on the other hand, could influence posttraumatic stress either directly or indirectly through posttrauma variables occurring after the trauma. Childbirth, like elective spine surgery, is a possibly traumatizing event that is highly predictable and therefore a suitable subject for research on the relevance of pre- and peritrauma risk factors in longitudinal studies (Garthus-Niegel, Von Soest, Vollrath, & Eberhard-Gran, 2013; König et al., 2016; van Son, Verkerk, van der Hart, Komproe, & Pop, 2005). While some studies (e.g. König et al., 2016; van Son et al., 2005) suggest both a vulnerability and a stress pathway, Garthus-Niegel et al. (2013) found that subjective experiences during birth are the most relevant predictor of PTSD symptoms.

## 2. Objective

In this paper, we focus on the interaction between pre- and peritrauma variables regarding posttraumatic stress. Patients undergoing elective spine surgery received questionnaires within one week before, one week after and three months after the potentially traumatic event ‘surgery’. Posttraumatic stress symptoms (PSS) prior to the surgery were investigated as a pretrauma variable, since posttraumatic stress symptomatology prior to the exposure of a new traumatic event was a strong predictor of posttraumatic stress in prior studies (Jubran et al., 2010; Kessler et al., 2014). Peritraumatic distress in general was analysed as a peritrauma variable, because peritraumatic distress has been shown to predict posttraumatic stress better than peritraumatic dissociation (Bui et al., 2010; Ladois-Do Pilar Rei et al., 2012). Our research question was whether peritraumatic distress mediates the relationship between pretrauma and posttrauma symptoms.

## 3. Method

### 3.1. Study design

This study is part of a prospective observational study including five pre- and postoperative time points. Assessments relevant for this research question were performed one week before the surgery (T0), one week after surgery (T1) and three months after the surgery (T2). Three questionnaires of those used in the original study were included in the present study, as described below. The study was approved by the medical Ethics Committee of the University of Regensburg.

### 3.2. Measures

The German version (Maercker, 1998) of the Posttraumatic Stress Scale (PTSS-10) (Weisæth, 1989) was used to operationalize pretrauma PSS at T0. It is a self-rating instrument covering PTSD symptoms in 10 questions and including symptoms of hyperarousal. Subjects rate the occurrence of symptoms in the last seven days on a scale of 0 (‘never’) to 3 (‘often’). The internal consistency varies between α = .85 and α = .91. The PTSS-10 has been used as a clinical research tool in various populations, for example, after traumatic experiences, after intensive care treatment and in populations of refugees and combat veterans (Maercker, 1998).

The German version (Maercker, 2002) of the Peritraumatic Distress Inventory (PDI) (Brunet et al., 2001), covering criterion A2 of PTSD in the DSM-IV, was applied to measure peritraumatic distress at T1. Regarding the surgery as a possibly traumatizing event, the patients’ experiences during the event can be assessed, such as negative emotions and perceived threat to life. The 13 items are rated on a Likert scale from 0 (‘not at all’) to 4 (‘extremely true’), resulting in a sum score of up to 52. According to Brunet et al. (2001), the reliability is *r* = .75–.76.

The German version (Maercker & Schützwohl, 1998) of the Impact of Event Scale–Revised (IES-R) (Weiss & Marmar, 1996) operationalized posttrauma PSS due to the surgery. It consists of 22 items covering three key symptoms of PTSD: intrusions, avoidance and hyperarousal. Patients rate their symptoms following the surgery on a 4-point Likert scale, ranging from 0 (‘not at all’) to 5 (‘often’) using only uneven numbers. A score for each of the three scales can be computed, or a total score by a regression equation can be computed, as proposed by Maercker and Schützwohl (1998). For this study, the IES-R sum score at T2 (three months after the surgery) and the T2 sum scores of the three subscales were statistically evaluated. The internal consistency is *r* = .90 for the scale intrusions, *r* = .71–.79 for avoidance and *r* = .90 for hyperarousal (Maercker & Schützwohl, 1998).

### 3.3. Participants

All patients enrolled in this study were undergoing elective spinal surgery at the neurosurgical department in the hospital Rechts der Isar, Munich, Germany, between March 2013 and December 2014. Elective spine surgeries are not medical emergencies but aim at improving functioning and correcting anatomical lesions. The sample included patients diagnosed with degenerative diseases or tumours: *N* = 231 patients gave informed consent to take part in the study. Of those patients, 38.5% (*n* = 89) provided data on posttraumatic stress symptomatology (PTSS-10) at T0, on peritraumatic distress (PDI) at T1 and on posttraumatic stress symptomatology due to the surgery (IES-R) at T2. The demographics of this sample are presented in Table 1. The included patients did not differ significantly from the drop-out patients in the investigated variables, except for the variable pretrauma PSS: posttraumatic symptomatology before the surgery (PTSS-10) was higher in the drop-out group than in the included patients (*t*(205) = 2.81, *p* = .01) with an effect size of Hedges, *g *= 0.39. The two groups did not differ in age (*t*(229) = 0.82, *p* = .41) or in variables such as gender (Fisher’s exact test (FET): *p* = 1.00), diagnosis (FET: *p* = .42) and marital status (FET: *p* = .54).

**Table 1.** Sample description.

Variables		Included patients	Drop-out
Age: *M* (*SD*)		58.13 (14.50)	59.72 (14.19)
PTSS-10 at T0: *M* (*SD*)		11.09 (6.96)	13.74 (6.51)
PDI at T1: *M* (*SD*)		7.87 (8.60)	
IES-R sum score at T2: *M* (*SD*)		16.39 (19.28)	
IES-R Intrusions score at T2: *M* (*SD*)		4.67 (7.06)	
IES-R Avoidance score at T2: *M* (*SD*)		5.94 (7.83)	
IES-R Hyperarousal score at T2: *M* (*SD*)		5.78 (6.60)	
Gender	Female: *n* (%)	37 (41.6)	60 (42.3)
	Male: *n* (%)	52 (58.4)	82 (57.7)
Diagnosis	Degenerative disease, spinal fusion: *n* (%)	60 (67.4)	85 (59.9)
	Degenerative disease, other procedure: *n* (%)	19 (21.3)	34 (23.9)
	Tumour: *n* (%)	8 (9)	21 (14.8)
	Other: *n* (%)	2 (2.2)	1 (1.4)
Marital Status	Single: *n* (%)	16 (18)	17 (12)
	Married: *n* (%)	61 (68.5)	99 (69.7)
	In a relationship: *n* (%)	8 (9)	14 (9.9)
	Widowed: *n* (%)	4 (4.5)	11 (7.7)

*M* = mean; *SD* = standard deviation.

### 3.4. Statistical analysis

**Figure 1.**
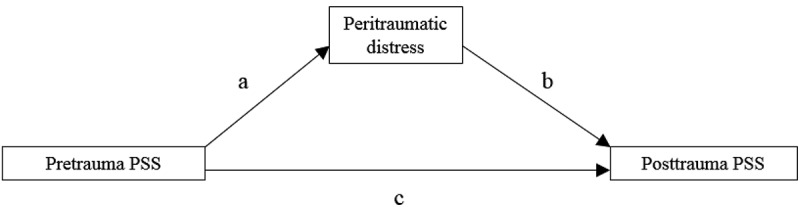
Path diagram of the mediation analysis. PSS = posttraumatic stress symptomatology.

SPSS 23 was used to perform the statistical analyses. Means (M), standard deviations (SD), percentages (%) and frequencies were computed as descriptive statistics. Fishers Exact Tests (FET) and *t*-tests for independent samples were performed for the drop-out analysis. Pearson correlation coefficients were performed to investigate the relationships between pretrauma PSS, peritraumatic distress and posttrauma PSS. To analyse whether peritraumatic distress mediates the relationship between pretrauma PSS and posttrauma PSS, the PROCESS macro was used (Hayes, 2013). Within PROCESS, we chose model 4 and 10.000 bias-corrected bootstrap samples. A 95% confidence level was chosen to apply a *p*-value of .05. PROCESS was used because it generates direct effects (effect of pretrauma PSS on posttrauma PSS) as well as bootstrapped indirect effects (effect of pretrauma PSS on posttrauma PSS through peritraumatic distress). All statistical tests were performed two-tailed. Correlations as well as mediation analysis were performed for IES-R sum score and its three subscales. In the mediation analyses, IES-R at T2 (sum score and subscale scores) were the outcomes, PTSS-10 at T0 functioned as predictor and PDI at T1 as mediator. Figure 1 displays the path diagram of the mediation analyses.

## 4. Results

First, we investigated how pretrauma PSS, peritraumatic distress and posttrauma PSS are correlated with one another. The results of this analysis are presented in Table 2.

**Table 2.** Correlations between pretrauma PSS, peritraumatic distress and posttrauma PSS.

	Pretrauma PSS (PTSS-10 at T0)	Peritraumatic distress (PDI at T1)
Peritraumatic distress (PDI at T1)	0.39**	-
Posttraumatic PSS (IES-R at T2)	0.34**	0.66**
Posttraumatic PSS – Intrusions (IES-R at T2)	0.25*	0.65**
Posttraumatic PSS – Avoidance (IES-R at T2)	0.31**	0.47**
Posttraumatic PSS – Hyperarousal (IES-R at T2)	0.34**	0.67**

** *p* < .001.

Second, the mediating effect of peritraumatic distress on the relationship between pretrauma PSS and posttrauma PSS was analysed. The results are summarized in Table 3. This analysis revealed that pretrauma PSS significantly predicted peritraumatic distress (path a estimate = 0.49, *p* < .001) and peritraumatic distress significantly predicted posttrauma PSS (path b estimate = 1.39, *p* < .001). By inspecting the bias-corrected bootstrap confidence intervals, it can be seen that the indirect effect of pretrauma PSS on posttrauma PSS through peritraumatic distress (path a x b estimate = 0.68) was different from zero (lower limit: 0.28; upper limit: 1.21). The direct effect of pretrauma PSS on posttrauma PSS was not statistically significant (path c estimate = 0.26, *p* = .30).

**Table 3.** Results of the mediation analysis using IES-R sum score.

Normal theory test				
	Estimate	SE	*t*	*p*
Effect of pretrauma PSS on peritraumatic distress (path a)	0.49	0.12	3.99	< .001
Effect of peritraumatic distress on posttrauma PSS (path b)	1.39	0.20	7.07	< .001
Direct effect of pretrauma PSS on posttrauma PSS (path c)	0.26	0.24	1.05	.30
Bootstrap results for indirect effects				
	Estimate	SE	Lower	Upper
Indirect effect of pretrauma PSS on posttrauma PSS through peritraumatic distress (a x b path)	0.68	0.23	0.27	1.21

SE = standard error; PSS = posttraumatic stress symptoms.

Because, the direct effect was not significant, whereas the indirect effect reached significance, it can be concluded that the relationship between pretrauma PSS and posttrauma PSS was fully mediated by peritraumatic distress. When the IES-R subscales functioned as outcomes, the mediation analyses yielded comparable results, i.e. the indirect effect became statistically significant and the direct effect did not attain statistical significance anymore (see Tables 4–6).

**Table 4.** Results of the mediation analysis using IES-R subscale Intrusions.

Normal theory test				
	Estimate	SE	*t*	*p*
Effect of pretrauma PSS on peritraumatic distress (path a)	0.49	0.12	3.99	< .001
Effect of peritraumatic distress on posttrauma PSS (path b)	0.53	0.07	7.26	< .001
Direct effect of pretrauma PSS on posttrauma PSS (path c)	< 0.01	0.09	−0.03	.98
Bootstrap results for indirect effects				
	Estimate	SE	Lower	Upper
Indirect effect of pretrauma PSS on posttrauma PSS through peritraumatic distress (a x b path)	0.26	0.08	0.11	0.45

SE = standard error; PSS = posttraumatic stress symptoms.

**Table 5.** Results of the mediation analysis using IES-R subscale Avoidance.

Normal theory test				
	Estimate	SE	*t*	*p*
Effect of pretrauma PSS on peritraumatic distress (path a)	0.49	0.12	3.99	< .001
Effect of peritraumatic distress on posttrauma PSS (path b)	0.37	0.09	4.06	< .001
Direct effect of pretrauma PSS on posttrauma PSS (path c)	0.17	0.11	1.45	.15
Bootstrap results for indirect effects				
	Estimate	SE	Lower	Upper
Indirect effect of pretrauma PSS on posttrauma PSS through peritraumatic distress (a x b path)	0.18	0.08	0.06	0.40

SE = standard error; PSS = posttraumatic stress symptoms.

**Table 6.** Results of the mediation analysis using IES-R subscale Hyperarousal.

Normal theory test				
	Estimate	SE	*t*	*p*
Effect of pretrauma PSS on peritraumatic distress (path a)	0.49	0.12	3.99	< .001
Effect of peritraumatic distress on posttrauma PSS (path b)	0.48	0.07	7.25	< .001
Direct effect of pretrauma PSS on posttrauma PSS (path c)	0.09	0.08	1.10	.27
Bootstrap results for indirect effects				
	Estimate	SE	Lower	Upper
Indirect effect of pretrauma PSS on posttrauma PSS through peritraumatic distress (a x b path)	0.23	0.08	0.10	0.41

SE = standard error; PSS = posttraumatic stress symptoms.

## 5. Discussion

Correlational analysis confirms associations between pre-, peri- and post-trauma factors, meaning that pre- as well as peritraumatic factors are linked to the development of PTSD symptomatology after surgery. The correlations can be interpreted as following: people who are more distressed by PTSD symptoms before surgery exhibit more symptoms of PTSD three months after surgery. Moreover, when experiencing greater distress by PTSD symptoms before surgery, patients perceive the procedure as more stressful, and those with higher peritrauma distress suffer from more PTSD symptoms three months after the surgery.

Even though peritraumatic distress seems to be more important than preoperative distress in predicting posttrauma symptoms, as this correlation is the strongest observed, it is only when the mediation analysis is applied that its impact can be seen clearly. Not only is the linkage between peritraumatic distress and PTSD symptoms the strongest, as found in other studies (Brewin et al., 2000; Kessler et al., 2014; Ozer et al., 2003; Trickey et al., 2012), but also the relationship between pre- and posttrauma PSS is no longer significant when peritraumatic distress is controlled for as mediating factor. This effect could be shown for main symptom clusters of PTSD, intrusions, avoidance and hyperarousal, as well as general PSS, proving that no over- or undermodulation on symptom level occurs. Peritraumatic factors are subject to controversial discussions, as opinions differ whether they are influential at all (O’Donnell, Creamer, McFarlane, Silove, & Bryant, 2010) and whether peritraumatic distress or dissociation has a stronger impact on the development of PTSD (Fikretoglu et al., 2006). In the DSM-5, the criterion A2 – describing the experience of intensive fear, helplessness or horror during the trauma – was removed. In contrast to this, the study at hand shows the importance of those peritrauma experiences for the development of PTSD symptoms. Even if peritraumatic distress is not included as diagnostic criterion, its relevance as risk factor should be paid attention to (Karam et al., 2010). In the study by Garthus-Niegel et al. (2013), comparable results were found, using a similar design in another population. Childbirth as well as spine surgery, among other medical conditions, can serve as research opportunities to study general mechanisms in the development of PTSD.

Mediation analysis can provide useful new insights into risk factors for the development of PTSD reported in various studies. As soon as a relationship between two variables is established, questions arise concerning the mechanisms and conditions of that relationship. Mediation analysis can answer questions on how an effect operates (Hayes, 2013).

Various restrictions of the study have to be denoted when interpreting the results. Based on the study by Kessler et al. (2014), posttraumatic symptomatology before the potential trauma, namely the surgical procedure, was considered a risk factor. The short screening instrument used in this study, the PTSS-10, can be criticized for containing a range of symptoms not specific for PTSD, such as sleeping problems. In general, questionnaires may not cover all aspects of a diagnosis and therefore may not be applicable as a diagnostic tool (Jackson et al., 2007). Consequently, the PTSS-10 could be seen rather as a measure of general psychological distress caused by the anticipation of the upcoming surgery and complications during the procedure. Moreover, the consequences of the patients’ conditions, like pain, various treatments or handicaps, are possibly distressing as well. On the other hand, the PTSS-10 and all the measures of this study are psychometrically sound screening instruments that are especially helpful when quick orientation is needed (Nickel et al., 2004).

In our study, possible confounds, such as gender, age, duration of surgery or depression, were not analysed due to the small sample size. Moderated mediation analyses (Hayes, 2013; Probst et al., 2016) are needed in the future to evaluate whether the contribution of pre- and peritraumatic factors on the development of PTSD symptoms is different for e.g. different age groups.

High drop-out rates may also restrict generalizability. Results have to be treated with care. High drop-outs are unfortunate yet common in longitudinal studies, such as König et al. (2016) and van Son et al. (2005). We did not ask for dropout reasons and recommend to do so in future studies. Nevertheless, the study and the dropout sample only differed in one variable, the level of pretrauma PSS. This difference is relatively small (effect size g = .39). T

he longitudinal design of this study is a crucial strength since previous mediation analyses were often cross-sectional (Lee et al., 2015; Probst, Pryss, Langguth, & Schlee, 2016).

Besides strengths and limitations of this specific study, the concept of PTSD in medical settings is a controversial matter (Rosen, Spitzer, & McHugh, 2008). Being affected by an illness and being exposed to a possibly traumatic experience is a rather new subject of interest. It is hard to define which aspect of it causes posttraumatic stress, as a medical condition itself can be seen as a psychological burden (Kangas, Henry, & Bryant, 2002). As rather low levels of PTSD in this study indicate, elective spine surgery may not be perceived as a traumatic experience by most patients. Peritraumatic distress in medical and specifically neurosurgical settings differs from experiences e.g. during a natural catastrophe or combat situations. In the DSM-5 (American Psychiatric Association, 2013), only a few situations in medical settings are included as traumatic: ‘A life-threatening illness or debilitating medical condition is not necessarily considered a traumatic event. Medical incidents that qualify as traumatic events involve sudden, catastrophic events (e.g. waking during surgery, anaphylactic shock)’ (p. 274). According to Rosen et al. (2008): ‘Criterion A events are neither necessary nor sufficient to produce PTSD. Instead, they appear to represent high-magnitude stressors that are otherwise indistinct from the full range of stressors that can have an impact on an individual and create risk for psychiatric morbidity’ (p. 3).

## 6. Conclusions

This study adds important evidence to the controversial discussion on risk factors for PTSD in medical settings. The finding that patients who are distressed preoperatively show more symptoms of PTSD after surgery can only be understood when patients’ reactions, emotions and thoughts during or shortly before and after surgery are taken into account. To be able to generalize such results, they need to be researched and replicated in other settings. For spinal surgeons specifically, our study shows that peritraumatic distress and the subjective stress patients experience due to the surgery has to be taken seriously. Assessing this distress in a short questionnaire after surgery can help identifying patients at risk of developing symptoms of PTSD. Consequently, those patients should be considered for psychological treatment in order to ensure not only mental health but also optimal recovery.
